# CREATING A PORTAL PLUS

**DOI:** 10.1093/geroni/igz038.2221

**Published:** 2019-11-08

**Authors:** Linda R Phillips, Beverly Heasley, Janice Crist, Cheryl Lacasse, Lorraine Martin-Plank, jian liu

**Affiliations:** 1 University of Arizona, Tucson, Arizona, United States; 2 Enhancing Lives, LLC, Tucson, Arizona, United States

## Abstract

Patient portals are popular health care tools. Few portals target caregiving families and few use predictive modeling to assist caregiving families make decisions about elder care options. Our goal is to incorporate the algorithms being developed for predicting tipping points in elder care to assist caregiving families visualize changes in older adults’ activity patterns, understand possible implications for continued in-home safety, and access educational information and consultation. While many patients use portals, use is restricted among persons in racial and ethnic minorities, with lower educational levels and poorer health literacy, and with fewer economic resources. The following strategies help us address these issues. Our portal archives data from affordable, wearable technology. Portal access uses similarly technology. Our educational materials use techniques known to be appealing and effective with older individuals in unique groups, e.g., results displays; fotonovelas. Input from community members/users helps maintain sensitivity to cultural appropriateness and health literacy.

